# Female infertility and diet, is there a role for a personalized nutritional approach in assisted reproductive technologies? A Narrative Review

**DOI:** 10.3389/fnut.2022.927972

**Published:** 2022-07-22

**Authors:** Amira Kohil, Spyridon Chouliaras, Shaikha Alabduljabbar, Arun Prasath Lakshmanan, Salma Hayder Ahmed, Johnny Awwad, Annalisa Terranegra

**Affiliations:** ^1^Research Department, Sidra Medicine, Doha, Qatar; ^2^Reproductive Medicine Unit, Sidra Medicine, Doha, Qatar

**Keywords:** diet, fertility, female reproductive health, art, nutrigenetics, nutrigenomics, nutriepigenome, microbiome

## Abstract

Female infertility is a major public health concern and a global challenge. It is a disorder of the reproductive system, defined as the inability to achieve a clinical pregnancy. Nutrition and other environmental factors are found to impact reproductive health in women as well as the outcome of assisted reproductive technologies (ART). Dietary factors, such as polyunsaturated fatty acids (PUFA), fiber as well as the intake of Mediterranean diet appear to exert beneficial effects on female reproductive outcomes. The exact mechanisms associating diet to female fertility are yet to be identified, although genomic, epigenomic, and microbial pathways may be implicated. This review aims to summarize the current knowledge on the impact of dietary components on female reproduction and ART outcomes, and to discuss the relevant interplay of diet with genome, epigenome and microbial composition.

## Introduction

According to the WHO, infertility is a global health issue affecting around 48 million couples around the world (1). Infertility is a disorder of the reproductive system defined as the inability to develop clinical pregnancy after 1 year of unprotected sexual intercourse (1). It is estimated that up to 15% of reproductive-aged couples worldwide are affected (1). Despite recent scientific advances and increased access and use of Assisted Reproductive Technologies (ART) globally, the overall burden of infertility has not shown any decline over the last two decades. Indeed, although *in vitro* fertilization (IVF) has revolutionized the landscape of infertility treatment, it remains far from being a panacea and success rates over the recent years have plateaued (2). The increasing prevalence of female impaired fecundity along with the high financial costs of fertility treatments and their relatively low success rates, make the identification of potentially modifiable factors and non-pharmacological treatments that can positively influence fertility-related outcomes, a pressing need. As different nutritional factors are found to affect female reproductive health and the potential success of ART, great interest is growing in understanding the relationship between them and the relevant research (3–7). The study of human fertility and fecundity is nonetheless very challenging. Therefore, most researchers focus on surrogate measures, such as time to intended pregnancy, pregnancy loss, or reproductive outcomes in women undergoing ART. Other commonly used fecundity parameters include reproductive hormonal profiles and markers of ovarian reserve such as antral follicle count and anti-Müllerian hormone (AMH). Admittedly, there is an inherent difficulty in studying the potential effect of diet and nutrition in infertility, which is anyway unique to any other pathology or condition as it is affected by the status of two rather than one individual- due to the multifactorial and extremely complex nature of reproduction (8). To complicate matters further, there is significant evidence that environmental factors may lead to direct toxicity, hormonal disruption and reactive oxygen species (ROS) production, demonstrating a causal association with female reproductive disorders (9). In today's world, food is not only a means to obtain nutrients, but invariably contains non-nutritive chemical substances which may occur either as environmental contaminants or are synthetic byproducts of food processing and packaging (7). Taking into consideration all the above constraints, we aim to present and appraise the available literature as a narrative review on the effects of diet on female reproduction. In addition, we will review the current knowledge on the impact of nutritional factors on the genome, epigenome and microbial composition, and explore associations with female reproductive health. The topic of diet and male infertility will not be addressed in this review, although the influence of nutrition and environmental factors on male fertility appears to be equally significant. We quote separately the available evidence and studies focusing either generically on women's reproductive health and various reproductive markers or on those women undergoing treatment with ART where the outcomes are more reproducible as we believe that these two categories have distinct characteristics that can draw interesting conclusions and comparisons. Finally, we assembled 3 tables summarizing the available evidence (Tables 1–**3**). Due to the variability of studies and outcomes and the small number of interventional studies, providing specific recommendations is particularly challenging. However, dietary patterns that improve reproductive outcomes do emerge and the interplay with the gene function could offer a valuable and more precise guide to women and health professionals alike.

**Table 1:** The effect of several nutritional factors and dietary patterns on female reproductive health.

	**Female reproductive health**		
**Nutrients**	**Study type/population**	**Findings**	Evidence*
Proteins	- Cohort study/Women reporting ovulatory infertility (10)	- Animal proteins increased the risk of ovulatory disorders (10)	**+**
	- Animal study/Mice (11)	- Low dietary protein improves fertility rate (11)	**±**
	- Cross sectional study/Women cohort (12)	- High soy intake decreases likelihood of pregnancy (12)	**+**
Carbohydrates	*Sugars:* - Cohort study/Premenopausal women (13) - Animal study/Lactating cattle (14)	*Sugars:* - High sugar intake is associated with infertility and low rate of pregnancy development (13, 14)	**++**
	*Fibers:* - Cohort study/Couples attempting to censive (15) - Cohort study/Women (18–44 years) (16)	*Fibers* intake shows contrasting data: - Increased chance of conception (15) - Decreased concentration of reproductive hormones and increased risk of anovulation (16)	**±**
Fats	*PUFA:* - Animal study/Female mice (17) - Cohort study/Women undergoing (IVF/ICSI) (18) - Cohort study/Women (18–44 years) (19)	*PUFA:* - The intake improves oocyte quality and embryo implantation rates (17, 18) - Seafood ω-3 PUFA are associated with increased progesterone concentrations (19) and DHA is associated with decreased risk of anovulation (19)	**++**
	*Trans-fatty acids:* - Cohort study/Premenopausal women (20) - RCTs/Overweight women (21), healthy men (22)	*Trans-fatty acids:* - Increased intake is associated with an increased risk of infertility (20) - Increased intake shows a negative impact on ovarian function as result of insulin resistance and increased inflammation (21, 22)	**++**
Vitamins	*Multivitamins:* - Cohort study/Premenopausal women (23)	*Multivitamins:* - Supplementation inversely correlates with ovulatory dysfunction in women (23)	**+**
	*Folate:* - Cohort study/Premenopausal women (24)	*Folate:* - Supplementation is associated with increased luteal progesterone levels and decreased risk of sporadic anovulation in premenopausal women (24)	**++**
	*Vitamin D:* - Animal study/Rats (25) - RCT/Women with history of pregnancy loss (26) - Cohort study/Healthy women (27)	*Vitamin D:* - Deficiency is associated with reduced fertility rates (25) - Contrasting data show no association between vitamin D and female fertility (26, 27)	**±**
Minerals	*Iron:* - Cohort study/Women with history of infertility (28)	*Iron:* - Supplementation is associated with decreased risk of ovulatory infertility (28)	**+**
	*Zinc:* - Animal studies/Rats (29), rhesus monkey (30) - Cohort study/Nulliparous women (31)	*Zinc:* - Deficiency results in abnormal menstrual cycles, longer time interval to pregnancy and decreased pregnancy rates (29–31)	**++**
	*Selenium:* - Cohort study/Nulliparous women (31)	*Selenium:* - Low serum concentration is associated with increased risk of subfertility and longer time interval to pregnancy (31)	**+**
Dietary pattern	*MedDiet**:*** - Cohort study/Healthy women (32) - Cross sectional study/Healthy women (33)	*MedDiet**:*** - Decreased risk of ovulatory disorders (66%) and other infertilities (27%) (32) - Improved ovarian morphology and ovarian function (33)	**++**
	*Western-like diet:* - Animal study/Macaques (34)	*Western-like diet:* - Low follicle survival rate and androstenedione and estradiol levels (34)	**+**

* + limited evidence; ++ moderate evidence; ± conflicting evidence.

IVF, *in vitro* fertilization; ICSI, intracytoplasmic sperm injection; PUFA, Polyunsaturated fatty acids; DHA, Docosahexaenoic acid; RCT, randomized controlled trial.

## Influence of diet on reproductive health in women

### Proteins

The Nurses' Health Study II (NHS II) suggested that healthy women who consumed animal-based proteins were at increased risk of ovulatory disorders compared to those who received plant-based proteins (10), which indicates a putative effect of the source of dietary protein on female reproductive health. Diets enriched with proteins derived from plant sources could influence favorably insulin sensitivity and Insulin-like Growth factor (IGF-I) levels as observed in other conditions (88–90). Moreover, studies conducted on ruminants showed that a high intake of protein affects ovarian activity through non-Luteinizing hormone (LH) mediated pathways (91), including the ovarian IGF system, which enhances intrafollicular IGF leading to increased follicle-stimulating hormone (FSH) sensitivity (92). Low dietary protein intake in mice resulted in improved fertility rates and controlled the ovarian primordial follicle reserve via Fibroblast Growth Factor 21 (FGF21) pathway (11).

Recently, protein intake and its effect on female reproductive health have received great attention due to its possible association with increased levels of environmental contaminants. Red meat and seafood, which are considered important protein sources, may have elevated levels of different contaminants, such as antibiotics, dioxins, mercury, and organochlorines (4, 93, 94).

Soy is another source of protein which has been under scrutiny due to its potential interference with the mechanisms of reproduction. It is enriched with phytoestrogens, which are potential reproductive disruptors as previously described in animal models (95, 96). There are few human studies evaluating the effects of soy on female reproductive health. However, a cross-sectional study found that women with a high intake of soy isoflavones were 13% less likely to achieve a pregnancy compared to women with a standard intake (12).

Conversely, the reduced intake of animal proteins, such as in the vegan diet, can cause the deficiency of important micronutrients, such as vitamin B12, zinc, calcium, and selenium (97). Particularly, the deficiency in vitamin B12 was observed in a cross-sectional study on Australian women in child-bearing age (18–44 years) that can negatively affect women reproductive health (98).

Overall, the exact influence of protein intake on female reproductive health is yet to be determined due to inherent difficulties in defining the impact on ovulatory function and fertility-related outcomes as well as the great variability of different protein sources (Table 1).

### Carbohydrates

There are limited studies evaluating the effect of carbohydrate intake on ovulatory function and fertility rate in healthy women. Although a diet low in carbohydrates has been shown to be associated with low insulin levels and improved pregnancy outcomes in women with polycystic ovarian syndrome (PCOS), this relationship has not been fully explored in healthy women (99).

The glycemic index and load are important indicators of the effect of a carbohydrate diet on blood glucose levels (100). Chavarro et al. found that higher dietary carbohydrates and glycemic load were associated with an increased incidence of infertility in healthy women (13). One may postulate that increased insulin levels resulting in elevated IGF-I and androgen levels (101) could create endocrine conditions similar to those associated with PCOS (102). Alternatively, high carbohydrate intake may occur at the expense of natural fats, which are prerequisite for the maintenance of ovulatory function (13, 103).

Simple sugars have also been associated with adverse effects on the reproductive performance of women, leading to a lower fertility potential. A diet rich in sugars was also associated with a lower likelihood of pregnancy in dairy cows (14). The biological mechanisms underlying these observed associations are yet to be identified. When female rats were fed a diet rich in sugar (sucrose 16%) followed by 1-day starvation, Sadowska et al. observed an increased glutathione peroxidase activity and malondialdehyde concentration and decreased activity of catalase in the uterus as well as decreased glutathione peroxidase and catalase activity and malondialdehyde concentration in the ovaries. The authors suggested changes in the oxidative balance as a plausible cause for fertility problems (104).

On another note, increased intake of non-digested carbohydrates, such as soluble fibers, was positively associated with the likelihood of conception. Unlike simple carbohydrates, the beneficial influence of a high fiber diet on female reproduction could be mediated by its effect on lowering blood glucose and thus resulting in a low dietary glycemic load and index (15). However, Gaskins et al. found that a high dietary fiber intake was associated with a reduction in FSH, LH, progesterone and estradiol levels, in addition to an increase in anovulatory cycles (16). A lower fermentation rate of fibers is known to promote gut microbial growth with health benefits for the host (105) and it can exert benefic effects on female reproductive health and ART outcomes, as discussed below. A summary of the evidence about carbohydrates intake is provided in Table 1.

### Fats

Different studies showed that fatty acids are needed in early reproductive events, such as oocyte maturation and embryo implantation (106, 107). It has been reported that diets rich in fatty acids may affect fertility and pregnancy rates positively by enhancing the production of steroids and prostaglandins (108). Polyunsaturated fatty acids (PUFA) intake appears to have beneficial effects on female reproduction, namely on oocyte quality and embryo implantation (17, 18). The type and amount of PUFA may have direct effects on ovarian steroid synthesis, oocyte maturation and pregnancy outcome (109–112). Marine ω-3 fatty acids for instance were associated with increased progesterone concentrations, while docosapentaenoic acid (DPA) was correlated with decreased anovulation (19). The underlying biological mechanisms could be explained by an effect on steroidogenesis and prostaglandin synthesis (113).

In contrast, trans-fatty acids were found to negatively impact ovulatory function in women by promoting insulin resistance (21, 114). A negative association with fertility was also supported by the NHS-II study (20). Possible mechanisms may involve binding to peroxisome proliferator-activated receptor (PPAR) and thus modulating its expression (115). PPAR is known to be implicated in different ovarian functions such as follicular rapture, corpus luteum formation and steroid hormones metabolism (116–118). Increased intake of trans fatty acids also leads to insulin resistance and an increase in inflammatory markers (21, 22).

Overall, despite numerous study limitations, the bulk of the evidence favors a negative effect of a diet that is rich in trans fatty acid and low in PUFA on the reproductive outcomes in healthy women (6) (Table 1).

### Micronutrients

Vitamins and minerals are essential dietary components that are implicated in different catabolic and anabolic processes associated with female reproduction (119). According to the NHS-II cohort study, multivitamin supplementation is inversely related to anovulatory dysfunction in women (23). Folate plays an essential role in human reproduction by affecting the synthesis of DNA, amino acids and methionine (120). The BioCycle study showed that intake of synthetic folate was associated with increased luteal progesterone levels and decreased risk of sporadic anovulatory cycles in premenopausal women (24). Vitamin D is another micronutrient implicated in the physiology of female reproduction, with receptors expressed in the ovaries, endometrium and placenta (121–123). A vitamin D deficient diet was associated with reduced fertility rates in female rodents (25). The same study showed restored reproductive capacity following vitamin D supplementation (25). It should be noted however, that these findings were not supported by other studies (26, 27).

Studies evaluating the effects of minerals on ovulatory infertility reported favorable outcomes following iron supplementation (28). Zinc deficiency was associated with abnormal menstruation and decreased pregnancy rates (29, 30). Women with low serum concentrations of zinc and selenium were more likely to experience longer time intervals to pregnancy (31). Deficiency of animal source nutrients, impacting levels of minerals such zinc, calcium and selenium, has been observed in women following vegan diet that could pose a risk for reproductive health (98).

Additional research is needed to further understand the role of different micronutrients on female reproductive health and thus shed light on the use of specific targeted nutrition as a therapeutic tool (Table 1).

### Dietary patterns

Dietary patterns are important practical nutritional tools that provide insight into the individual's dietary habits. Dietary patterns are implicated in multiple complex nutritional interactions that affect general health and wellbeing (124). Mediterranean dietary patterns and Western-style diets are found to have a positive and a negative effect on female reproductive health outcomes, respectively (32–34).

Chavarro et al. evaluated the influence of dietary patterns on female fertility. They described the optimal “fertility diet” to resemble very closely the Mediterranean dietary pattern. Such a diet, characterized by a high vegetarian to animal protein ratio, full-fat dairy products, decreased glycemic load, vitamins, and increased monounsaturated fatty acids (MUFA) to trans saturated fats ratio, was associated with a 66% reduction in ovulatory disorders and a 27% decrease in infertility risks (32). The same group of authors also showed that consumption of fruits and vegetables with high pesticide residues was associated with low rates of clinical pregnancy and live births after ART treatment (125). The pathway analysis showed that high pesticide exposure was associated with metabolic pathways involving vitamins, energy and enzyme metabolism (126). Kazemi et al. furthermore showed that the Mediterranean diet may improve ovarian morphology and ovarian function in women of reproductive age (33). The authors suggested an association between diet-related improvements in ovarian morphology and a decrease in insulin resistance, weight and hyperandrogenism (33).

In contrast, the Western dietary pattern prevalent in developed countries is characterized by a high intake of red meat, fat, sugar drinks and refined grains (127). Although linked to poor reproductive outcomes, the underlying mechanisms remain unclear. When female macaques were fed a Western-like diet, they expressed lower follicle survival rates and lower androstenedione and estradiol serum levels compared with controls (34). Further research is needed to elucidate potential molecular and cellular mechanisms associating dietary patterns to female reproduction (Table 1).

## The effect of diet on the outcomes of assisted reproductive technologies

### Proteins and ART

Similar to the effect of protein intake on general female fertility, various protein sources have been associated with different effects on ART success (35, 36). A cohort study showed that the intake of animal-sourced proteins (red meat) correlated negatively with embryo development and with the likelihood of clinical pregnancy after intracytoplasmic sperm injection (ICSI). The same study also showed that a higher intake of dietary elements enriched in ω-3 PUFA and other protein sources (fish and white meat) correlated positively with the likelihood of blastocyst formation (35). Women who consumed fish as a primary source of proteins had higher probability of live births after ART compared with controls (36). In addition, Braga et al. found that the consumption of animal-based proteins (red meat with 26–27% protein content) before IVF treatment was negatively associated with embryo development and pregnancy rates (35). This negative association could be explained by the increased absorption of advanced glycation end products (AGE), which are prone to be developed during the cooking process in animal-derived food rich in protein (128). It is suggested that AGE accumulation is associated with poor follicular and embryonic development as well as with a low likelihood of pregnancy due to intracellular damage by cross-linking, molecular trapping or binding to specific cell surface receptors (129, 130).

Interestingly, studies investigating the effect of the soy intake showed contrasting findings (12, 37, 40). The intake of soy isoflavones was found to be associated with a 13% higher live birth rate (age adjusted) in the EARTH study (37). Similarly, a positive association was established between the isoflavone supplementation and progesterone concentration, endometrial thickness and pregnancy rates in women undergoing infertility treatment (38). The same findings were observed following intrauterine insemination (IUI) and IVF treatments, in which increased endometrial thickness as well as pregnancy rates were also observed (39, 42). On the contrary, a study conducted by Jacobsen et al. found a negative association between isoflavone intake and the likelihood of pregnancy (12). Similarly, a study conducted by Chavarro et al. found that urinary bisphenol A (BPA) concentration was correlated with lower rate of implantation, pregnancy and birth rates in women not consuming soy (40). However, no relation was observed between these outcomes and BPA levels in women consuming soy (40). Finally, no associations were observed between phytoestrogens intake and female fecundability (41).

### Carbohydrates and ART

It is well-known that glucose homeostasis and insulin sensitivity are both affected by the type and amount of dietary carbohydrates, which further influence ovarian androgen production and function (15, 101). The intake of a hypocaloric diet (low glycemic index and glycemic load) was found to be positively associated with reproductive outcomes, such as the number of oocytes retrieved and live births in obese infertile women undergoing IVF and increased spontaneous pregnancy (43). This observed beneficial effect could be mediated through a reduction in body mass index (BMI), leptin concentration and body fat percentage (43). In another study, a high carbohydrate as well as fiber intake and a high dietary glycemic load were associated with poor ovarian response (44). However, an Italian cohort study found no significant associations between carbohydrate intake, dietary glycemic load/index and dietary fiber with IVF outcomes (45). Instead, complex carbohydrate intake, such as whole grain, was positively associated with the birth rate in women undergoing IVF, which could possibly be mediated through its effect on enhancing the endometrial thickness and endometrial receptivity (131).

### Fats and ART

Fatty acids, mainly PUFA, play an important role in the development and maturation at different reproductive stages, thus influencing fertility and its related outcomes in women (106, 107). During oocyte maturation and early embryo development, they act as energy substrates as well as precursors for prostaglandins and steroid hormones synthesis, which impact pregnancy maintenance (106, 107). The beneficial effects of dietary PUFA intake on female reproductive endpoints were also reported in ART settings. Moran et al. found that the intake of PUFA (ω-6 PUFAs and ω-3 PUFA) was associated with improved pregnancy outcomes in obese and overweight women undergoing IVF treatment (46). Moreover, a significant association was observed between the intake of ω-3 PUFA, estradiol (E2) levels, embryo morphology, and the number of follicles in these women (18). The EARTH cohort showed similar findings, where the intake of long-chain ω-3 PUFA was positively associated with the likelihood of birth rates (47). A positive association was also observed in women undergoing ICSI between follicular fluid and serum levels of specific PUFA (linolenic and linoleic acids) which cannot be synthesized by the body (132). A 6-weeks intervention trial using daily supplement with ω-3 PUFA and vitamin D in 56 couples undergoing IVF or ICSI showed no difference in the second cell cycle (CC2) but faster fourth cell cycle (CC4) and a significantly shorted third cell cycle (S3) improving embryo quality (48). It should also be noted that several other studies showed conflicting results with a negative association between elevated level of different PUFA and ART outcomes (49–51). These discrepancies can be attributed to the genetic variation among different ethnic backgrounds in fatty acid metabolism plus the complexity of PUFA metabolism (47, 133). In addition, inconsistencies in the type of PUFA investigated in different research studies could explain this discrepancy, since ovarian follicles might metabolize specific fatty acids differently (50). This was suggested by Jungheim et al., who described a weak association between fatty acid concentrations in follicular fluid and serum, as well as differences in the levels of fatty acid types in the follicular fluid [linoleic (25%), oleic (31%), palmitic (27%) and stearic acids (12%)] (50). In contrast to the general beneficial effect of PUFA on ART outcomes, the intake of dietary trans fatty acid has been associated with a negative influence on reproductive health in women undergoing ART (51). A study conducted by Eskew et al. showed a negative association between the trans fatty acid index and IVF-related outcomes, such are fertilization and blastulation rate, but no association with the intake of ω-3 PUFA (51). The authors of this study suggest that the potential mechanism behind this observed effect may be multifactorial and could involve modulation of PPAR expression and glucose homeostasis (51, 115).

### Micronutrients and ART

In the same way that the intake of different micronutrients is found to positively impact female reproductive health, this association is also observed with ART outcomes (5, 52, 53). It has been reported that women adhering to a “pro-fertility” diet, which is characterized by the intake of vitamin D, B12, folic acid whole grains, soy foods, dairy, fruits and vegetables, have increased likelihood of live birth after ART (52). In addition, in a cohort of women eligible for IVF treatment, women who consumed folic acid have decreased concentration of homocysteine in follicular fluid and serum, and showed improved quality and increased maturity of oocytes (53). High folate intake in women undergoing IVF/ICSI was also associated with increased rate of implantation, clinical pregnancy and live birth rates (5).

Vitamin D is an important micronutrient that has been linked with female reproductive health (25) and ART outcomes (54) although the existing evidence is controversial. In one study, women who were undergoing IVF and had higher levels of vitamin D (in both follicular fluid and serum) were found to have an impressive 4-folds higher pregnancy rate compared with vitamin D deficient women, thereby, suggesting a strong beneficial effect of vitamin D supplementation on ART outcomes (54). A systematic review also showed a positive relationship between vitamin D and rate of live births in women undergoing ART, suggesting that vitamin D supplementation could be a possible therapeutic tool (134). In addition, higher rates of clinical pregnancy per IVF cycle are observed in women with sufficient vitamin D level (54.9%) compared to those with vitamin D insufficiency (34.7%) (55). Similarly, a study conducted by Rudick et al. found that women undergoing IVF treatment and vitamin D deficient have a lower rate of clinical pregnancy development and lower birth rates (56). The authors of this study suggested that the impact of vitamin D on IVF could be mediated through a direct effect on the endometrium (56). In another group of women undergoing IVF and elective single embryo transfer (ESET), vitamin D deficiency was also found to be negatively correlated with clinical pregnancy rates (57). However, a number of studies have reported no association between vitamin D and fertility-related outcomes in women undergoing ART (58–60). The exact cause-effect relationship between vitamin D and ART outcomes is yet to be identified due to the high heterogeneity observed among the different observational and interventional studies (135).

Last but not least, various studies have shown that women undergoing IVF have reduced levels of several trace elements, mainly selenium, copper and zinc, in the follicular fluid, which impact the quality of the microfollicular environment (61–63). However, the level of these trace elements is restored after dietary supplementation and therefore indicates its possible role in improving fertility rates and ART outcomes (61–63). Albeit another study conducted by Ng et al. didn't find any association between the concentration level of zinc, calcium and copper in the follicular fluid with follicle volume, oocyte fertilization rate and a number of oocytes in the follicle in women undergoing IVF. These mixed results likely stem from the inherent difficulties of assessing reproductive outcomes in the infertile population (64).

### Dietary pattern and ART

Several studies have shown the potential positive impact of adherence to a Mediterranean diet in women undergoing IVF (65). Vujkovic et al. showed that adherence to the “Mediterranean” dietary pattern led to a 40% increased probability of achieving pregnancy after IVF/ICSI treatment (65). The adherence to this diet was reflected by relatively high concentrations of folate and vitamin B6 both in blood and in follicular fluid (65). Another study by Karayiannis et al. showed that adherence to the Mediterranean diet as assessed by using the validated MedDiet Score was associated with a 2.7-folds increase in clinical pregnancy and live birth rate in women aged <35. Interestingly the same effect was not evident in older women in the study (66). In a more recent investigation in women undergoing IVF, a positive association was found between adherence to the Mediterranean diet, fertilization rates and embryo yield (67). However, in this study Mediterranean diet was not correlated with clinical pregnancy and implantation rates (67). It is suggested that the Mediterranean dietary pattern is associated with improved ART outcomes as this pattern is rich in antioxidants, which are known to alleviate the oxidative damage on female reproductive health, as well as, being associated with improved fertility rates (67, 68). Another study conducted by Kermack et al. aimed to investigate the influence of pre-conceptional Mediterranean dietary intervention in women before IVF or ICSI treatment on key morpho-kinetic markers of early embryo development (48). The authors of this study observed that embryos derived from women consuming the “Mediterranean diet” (ω-3 PUFA, vitamin D and olive oil) have shortened the fourth cell cycle (CC4) and synchrony of the third cell cycle (S3) (48). Although several studies showed that the intake of the Mediterranean diet is associated with beneficial effects on female reproductive health and successful ART outcome, in an Italian cohort study of 474 women, adherence to the Mediterranean diet was not associated with favorable IVF outcomes (69). Another study showed that women undergoing IVF who adhere to the Dutch dietary recommendations (whole wheat bread, monounsaturated or polyunsaturated oils, vegetables, fruit and fish) have an 65% increased chance of ongoing pregnancy (136). On the other hand, the short-term intake of a Western-like diet was found to promote a proinflammatory intrafollicular microenvironment, which is associated with impaired preimplantation development in the female rhesus macaques model (70).

Overall despite the difficulties in evaluating dietary patterns and controlling the variable confounding factors it seems that nutritional modifications in subfertile women may positively affect fertility rates after ART and female reproductive health as a whole (137). A summary of the current evidence is presented in Table 2.

**Table 2:** The effect of several nutritional factors and dietary patterns on ART outcomes.

	**Assisted reproductive technologies**		
**Nutrients**	**Study type/population**	**Findings**	Evidence*
Proteins	*Animal proteins:* - Cohort study/Women undergoing ICSI (35) - Cohort studies/Women undergoing ICSI, ART (35, 36)	*Animal proteins:* - Red meat intake negatively correlates with embryo development and pregnancy rate (35) - Seafood sourced proteins increase likelihood of blastocyst formation and live births rate (35, 36)	**+**
	*Plant proteins:* - Cohort study/Women undergoing ART (37) - RCTs/Infertile women (38), Women undergoing IVF (39) - Cross sectional study/Women cohort (12) - Cohort studies/Women undergoing IVF (40), healthy women (41)	*Plant proteins (soy intake):* - Isoflavones supplementation increased pregnancy and live births rate, progesterone, endometrial thickness (37, 38, 42) - Soy intake shows no/reduced likelihood of pregnancy and female fecundability (12, 40, 41)	**±**
Carbohydrates	*Sugars:* - RCT/Obese and overweight infertile women (43) - Cross sectional study/Women eligible for IVF (44) - Cohort study/ Infertile couples eligible for IVF (45)	*Sugars:* - Low glycemic load diet is associated with the number of retrieved oocytes, spontaneous pregnancy, and live birth rate (43) - High glycemic load is associated with poor ovarian response (44) - No association is observed between dietary glycemic load and IVF outcomes (45) *Fibers:*	**±**
	*Fibers:* - Cross sectional study/Women eligible for IVF (44) - Cohort study/Infertile couples eligible for IVF (45)	- Poor ovarian response (44) - No association between fiber intake and IVF outcomes (45)	**±**
Fat	*PUFA:* - RCT/Overweight and obese women undergoing IVF (46) - Cohort studies/Women undergoing IVF and ICSI (18, 47) - RCT/Couples undergoing IVF and ICSI (48) - Cohort studies/Women undergoing IVF (49–51)	*PUFA:* - ω-3/ ω-6 PUFA intake is associated with improved pregnancy rates (46) - ω-3 PUFA is associated with estradiol (E2) levels, embryo morphology, number of follicles, pregnancy and birth rates (18, 47) - ω-3 PUFA combined with vitamin D supplementation improved embryo quality (48) - contrasting data show different PUFA associated with decreased chance of pregnancy development (49–51) and no association between ω-3 PUFA and IVF outcomes (51)	**±**
	*Trans-fatty acids:* - Cohort study/Women undergoing IVF (51)	*Trans-fatty acids:* - The intake negatively correlates with IVF fertilization rate and blastulation rate (51)	**+**
Vitamins	*Multivitamins:* - Cohort study/Women underwent ART (52)	*Multivitamins:* - The combination of multivitamins, whole grains, soy foods, dairy, fruits and vegetables (“pro-fertility diet”) is associated with an increased likelihood of live birth (52)	**+**
	*Folate:* - Cohort study/Women undergoing ART (5) - RCT/Women undergoing IVF (53)	*Folate:* - Intake is associated with improved ART outcomes (5, 53)	**++**
	*Vitamin D:* - Cohort studies/Women undergoing IVF (54–57) - RCT (58), cohort study (59) and cross-sectional study (60)/Women undergoing IVF	*Vitamin D:* - High serum level is associated with high pregnancy and live births rate (54–57) - Contrasting data show no association of vitamin D levels with pregnancy rate and number of collected fertilized oocyte (58–60)	**±**
Minerals	*Zinc:* - RCTs (61, 62), cohort study (63)/Women undergoing IVF - Cohort study/Women undergoing IVF (64) *Selenium and copper:* - RCTs (61, 62), cohort study (63)/Women undergoing IVF	*Zinc:* - The level of zinc is restored after dietary supplementation indicating its possible role in improving fertility rates and ART outcomes (61–63) - In contrast, no association between the level of zinc, calcium and copper and follicle volume, oocyte fertilization rate and number of oocytes (64) *Selenium and copper:* - Follicular level of selenium and copper impact the quality of the microfollicular environment (61–63)	**±**
Dietar patterns	*MedDiet:* - Cohort studies/Couples undergoing IVF and ICSI (65–68) - RCT/Couples undergoing IVF and ICSI (48) - Cohort study/Couples undergoing IVF (69)	*MedDiet:* - Increased fertilization rate, embryo yield, likelihood of pregnancy and live births rate (65–68) - Associated with embryos with shortened fourth cell cycle (CC4) and synchrony of the third cell cycle (S3) (48) - Contrasting study shows no association with IVF outcomes (69)	**±**
	*Western-like diet:* - Animal study/Rhesus Macaques (70)	*Western-like diet:* - Increased proinflammatory intrafollicular microenvironment and impaired preimplantation development (70)	**+**

* + limited evidence; ++ moderate evidence; ± conflicting evidence.

ICSI, intracytoplasmic sperm injection; IVF, *in vitro* fertilization; PUFA, polyunsaturated fatty acids; DHA, docosahexaenoic acid; ART, assisted reproductive technology; RCT, randomized controlled trial.

Equally physical activity and energy expenditure have an important role in fertility however the evidence is still contrasting on the efficacy of the physical activity and the difference according to the type of patients: increasing energy expenditure will benefit the normal population (138), where excessive exercise can be detrimental in female athletes (139). As many reviews are available on the topic and the argument (138–141), we do not discuss it further in this review.

## Nutrigenetics and nutrigenomics

Nutrigenomics is the science investigating the influence of nutritional factors on gene expression, epigenetics, protein expression, metabolome, and microbiome, while nutrigenetics studies the influence of genetic variations on nutrients metabolism (142, 143). The development of these sciences helped to shed light on the potential interplay between genes function and nutritional intake which can contribute to the establishment of more precise nutritional assessment tools (144). A better understanding of these interconnections may aid in the development of personalized nutritional recommendations for the purpose of optimizing fertility outcomes (Table 3).

**Table 3:** The role of nutrigenomics, nutri-epigenetics and diet - gut microbiome axis in influencing ART outcomes.

**Nutrients**	**Study type/population**	**Nutrigenomics and nutrigenetics**	**Nutri-epigenetics**	**Diet-gut microbiome**
Fats	*PUFA:* - Animal study/Mice (71) *SFA:* - Animal study/Mice (72)	- *PUFA* intake is associated with upregulation of genes involved in folliculogenesis (BMPER, FLT4, BDNF, CRHR1, AGT) ovulation (CCR3, PTX3, AGT), steroidogenesis (CRHR1, AGT, BDNF) (71) - *SFA* intake is associated with downregulation of the embryonic gene TWIST1 (73) and differential expression of ovulation genes (EDN2, TNFAIP6, ERRFIL, PRKG2, NFIL, EDN2 and NR4AL) (72)	No evidence available	No evidence available
Carbohydrates	- Animal Study/Drosophila melanogaster (74) - Cell Model/ Bovine fibroblast cell line (75)	High sucrose diet is associated with decreased expression of antioxidant and mitochondrial genes, impairing oocyte maturation (74)	Low carbohydrate intake increases levels of BOHB (76) that is associated with elevated H3K9ac level, leading to enhanced *FOXO3A* expression in the blastocyst (75)	No evidence available
Protein	- Cross sectional study/Women undergoing IVF (77)	No evidence available	No evidence available	Animal protein -derived TMAO is decreased in follicular fluid of oocytes developing high quality embryos (77)
Fibers	- RCT/Infertile women undergoing IVF (78)	No evidence available	No evidence available	Fiber intake is associated with increased abundance of Bifidobacterium, decreased abundance of Paraprevotella and Blautia and increased ART success rate (78)
Micronutrients	*Vitamin D:* - RCT/Infertile women candidate for IVF, women undergoing ART with vitamin D deficiency (79, 80) *Vitamins B:* - Animal study/Drosophila melanogaster (81) - Animal study/Black x Holstein F1 cows (82) *Folate:* - RCT/Women with history of miscarriage (83) - Animal study/Mice (84) *Selenium and Zinc:* - RCT/Women with PCOS candidate for IVF (85, 86) - Animal study/Mice (87)	*Vitamin D* supplementation improves glycemic profile via: - upregulation of PPAR-γ, GLUT-1, LDLR, VDR, GSTA3 and IL-21R (79, 80) - downregulation of PTGS2, AGER and RXRB (79, 80) *Folate* supplementation reduce homocysteine concentration in women homozygous for MTHFR-gene 677C > T (83) *Selenium* supplementation: - upregulation of VEGF, PPAR-γ and GLUT-1 (85, 86) - downregulation of IL-1, TNF-α and LDLR (85, 86)	*Vitamins B:* - *Biotin* deficiency is associated with a decrease in biotinylated histones and an increase in fertility rates (81) - *Vitamin B* mixture increases methylation level of H3K27 (82) *Folate:* - Reduced DNA methylation variance in *KCNQ1OT1, SNRPN* and *PEG1* (84) - increased global DNA methylation levels in both embryonic and placental tissues (84) *Zinc* deficiency is associated with decreased histone H3K4 and global DNA methylation in oocytes (87)	No evidence available

PUFA, Polyunsaturated fatty acids; BMPER, BMP Binding Endothelial Regulator; FLT4, Fms Related Receptor Tyrosine Kinase 4; BDNF, Brain-derived Neurotrophic Factor; CRHR1, Corticotropin Releasing Hormone Receptor 1; AGT, Angiotensinogen; CCR3, Chemokine Receptor 3; PTX3, Pentraxin 3; SFA, Saturated Fatty Acids; TWIST1, Twist-Related protein 1; EDN2, Endothelin 2; TNFAIP6, Tumor Necrosis Factor-Inducible Gene 6; ERRFIL, ERBB Receptor Feedback Inhibitor 1; PRKG2, Protein Kinase CGMP-Dependent 2; NFIL, Nuclear Factor, Interleukin 3; NR4AL, Nuclear Receptor Subfamily 4 Group A Member 1; BOHB, β-hydroxybutyrate; H3K9ac, Histone 3 Lysin 9 Acetylation; FOXO3A, Forkhed Box O3; TMAO, Trimethylamine-N-Oxide; ART, Assisted reproductive technology; PPAR-γ, Peroxisome Proliferator- Activated Receptors; GLUT-1, Glucose Transport; LDLR, Low-Density Lipoprotein Receptor; VDR, Vitamin D Receptor; GSTA3, Glutathione S-Transferase A3; IL-21R, Interleukin 21 Receptor; PTGS2, Prostaglandin-Endoperoxide Synthase 2; AGER, Advanced Glycosylation End-Product Specific Receptor; RXRB, Retinoid X Receptor Beta; VEGF, Vascular Endothelial Growth Factor; IL-1, Interleukin 1; TNF-α, Tumor Necrosis Factor Alpha; H3K27, Histone 3 Lysin 27; KCNQ1OT1, KCNQ1 Overlapping Transcript 1; SNRPN, Small Nuclear Ribonucleoprotein Polypeptide N; PEG1, Paternally Expressed Gene 1; H3K4, Histone 3 Lysin.

### Nutrigenetics and nutrigenomics in female reproduction and ART

Although female infertility is strongly affected by environmental factors, genetics play an essential part in its pathogenesis, in what appears to be a polygenic inheritance pattern (145). Both ovulatory dysfunction and endometriosis are recognized causes of female infertility and have been known to have familial predisposition indicating genetic involvement (146). In addition, different genetic abnormalities have been reported to cause female infertility, such as chromosomal alterations and single gene mutations [Anosmin 1 (*ANOS1)*, Gonadotropin Releasing Hormone *(GnRH)* and Homoebox *(HOX)* genes]. These genetic risk factors lead to the development of endometriosis, hypogonadism and ovarian dysfunction, which ultimately lead to infertility (146).

Genetic variations are known to impact also on nutritients metabolism and dietary requirements (147) and certain genetic variants can affect ART outcome. Two variants of the methylenetetrahydrofolate reductase (*MTHFR*) gene (c.677C > T and c.1298A > C) are well-known to reduce its enzymatic activity and increase homocysteine levels, which alter folate metabolism (148). A study conducted by Nelen et al. showed that daily supplementation with folic acid reduced homocysteine concentration in women homozygous for *MTHFR*-gene 677C > T mutation with a history of recurrent miscarriages (83). It appears that folic acid supplementation has a beneficial role in female patients with *MTHFR* polymorphisms. In their study, Laanpere et al. showed that folate-metabolizing gene polymorphisms may affect IVF outcomes, suggesting that heterozygotes rather than wild type homozygotes have overall more favorable ART outcomes including embryo quality and clinical pregnancy rates (149). Another study in women undergoing ICSI for male factor infertility showed that in women with the TT genotype for *MTHFR* (c.677C > T) serum AMH levels were increased however there was a non-significant trend toward lower clinical pregnancy rates compared to the ones with the CT genotype (150). Finally, a case-series report showed that couples carrying *MTHFR* variants (C677T or A1298C) suffer from fertility problems or failed ART, and they can benefit from 5-methyltetrahydrofolate (5-MTHFF) supplementation vs. folic acid (151). The study showed that high intake of folic acid in these patients resulted in un-metabolized folic acid syndrome. Moreover, the treatment with 5-MTHFF increased the chances of pregnancy development.

Nutrients have been known for a long time to act as dietary signals which affect gene expression, protein production and hormonal function (142). Macro- and micro-nutrients are able to control the transcription process. Flavonoids for instance facilitate transcriptional activation by binding to nuclear receptors leading to co-repressor dissociation and co-activator recruitment (142, 152). Irani et al. found that vitamin D supplementation has a beneficial effect on folliculogenesis in women with PCOS, by attenuating AGE-mediated inflammation (153).

Chronic consumption of high-fat diet (exceeding the 35% calories recommendation) (154), has been associated with metabolic and physiological disturbances involving the reproductive system. Ruebel et al. aimed to compare oocyte gene expression in overweight/obese women undergoing fertility treatment on the basis of their dietary intake of fats and carbohydrates compared to normal weight controls (73). They found a positive association between saturated fatty acid (SFA) intake and downregulation of the oocyte Twist Family BHLH Transcription Factor 1 (*TWIST1*) gene expression in overweight/obese women. No significant associations were found with BMI or adiposity, suggesting that maternal fat intake plays an important role in modulating the inflammatory response of the oocyte (73). *TWIST1* is a transcription factor involved in embryonic development and influences lipid metabolism and inflammation (155, 156). Increased fat intake, regardless of obesity status, was also found to be associated with menstrual irregularity and ovarian reserve depletion. The fat diet also altered the expression of genes controlling ovulation, luteinization and luteolysis (72). Another study investigated the effects of an acute dietary intake of docosahexaenoic acid (DHA), an ω-3 PUFA, on restoring ovarian gene expression following chronic high fat diet consumption in female mice (71). The investigator reported a beneficial outcome of DHA supplementation on the upregulation of ovarian genes involved in folliculogenesis, ovulation and steroidogenesis. The expression of the *Gm42508* genetic marker was nonetheless increased after high-fat feeding and was unaffected by DHA, which suggests a potential role in high fat diet-induced ovarian dysfunction (71). Oseikria et al. investigated the effects of DHA supplementation on oocyte maturation and embryo development in cattle undergoing *in vitro* maturation (IVM). Low dose DHA supplementation during IVM was associated with improved oocyte development without affecting lipid metabolism gene expression (157). Furthermore, in female rhesus macaques, the intake of Western-like diet was found to be associated with dysregulation of genes involved in mitochondrial function, protein channel activity, RNA binding and cell differentiation (70).

Increased intake of dietary sugars was also observed to negatively impact female reproductive efficiency and the expression of essential genes associated with fertility. A study conducted using Drosophila melanogaster as animal model found that female adults fed with a high sucrose diet developed insulin resistance, accumulated lipids in the ovaries and had impaired oocyte maturation. Decreased expression of antioxidant and key mitochondrial genes were also detected (74). This study also showed that a high sucrose diet-induced maternal obesity (158), and impacted negatively fertility, without nonetheless lending support for a direct effect of high sucrose intake (74).

Micronutrients (vitamins and minerals) are found to impact female reproductive health and play essential roles in different stages of conception (119). In a randomized controlled trial (RCT), Dastornia et al. investigated the effects of vitamin D supplementation on gene expression in PCOS patients who were candidates for IVF (79). They found an upregulation of *PPAR-*γ, Glucose transport (*GLUT-1)* and low-density lipoprotein receptor (*LDLR)* genes after vitamin D supplementation when compared to the controls (79). Another randomized controlled trial observed the upregulation of vitamin D receptor (*VDR*), glutathione S-transferase A3 (*GSTA3*) and interleukin 21 receptor (*IL-21R*), as well as the downregulation of prostaglandin-endoperoxide synthase 2 (*PTGS2*), advanced glycosylation end-product specific receptor (*AGER*) and retinoid X receptor beta (*RXRB*) in women receiving vitamin D supplementation compared to placebo (80). The supplementation improved the glycemic profile affecting the glycemic and lipid metabolism (79) and activating the antioxidant pathways (80). While selenium was also implicated in improving reproductive health, the exact mechanism is not yet identified (159). In a recent study conducted by Heidar et al. the investigators found the expression of interleukin-1 (*IL-1*) and tumor necrosis factor alpha (*TNF-*α) to be downregulated following selenium supplementation in PCOS women undergoing IVF. Conversely, vascular endothelial growth factor (*VEGF*) was upregulated in the same study population (85). The same investigators also found that selenium supplementation resulted in the upregulation of *PPAR-*γ and *GLUT-1*, along with the downregulation of *LDLR* (86).

### Nutri-epigenetics in female reproduction and ART

Nutrition has a strong impact on the epigenome during early embryo development. It also affects oocyte maturation and oocyte stores of mitochondria and metabolites (160), thus a better understanding of the interplay between diet and epigenetic modifications and its impact on female reproductive health is needed.

The relationship between nutritional factors, histone modification and fertility is not clearly understood. However, in a study by Sangalli et al there was a potential relationship between intake of β-hydroxybutyrate (BOHB), histone acetylases (H3K9ac) and embryonic development. The authors also found that BOHB has an epigenetic effect (enhancing the level of H3K9ac) in the early zygote stage that was maintained until the blastocyst stage and resulted in enhanced Forkhed Box O3 (FOXO3A) expression and blastocyst production (75). Increased BOHB level is associated with fasting and lower carbohydrate intake (76), thereby, this study shed the light on the potential interplay between maternal nutrition, histone modification and reproduction. Another study conducted on Drosophila melanogaster showed that flies maintained on a biotin-deficient medium are associated with a decrease in biotinylated histones and an increase in fertility rates (81). In addition, vitamin mixtures (vitamin B and vitamin B-like substances) are found to decrease the mRNA expression level of thioredoxin-interacting protein (*TXNIP*) and increase level of H3K27 trimethylation after IVF treatment of bovine embryos (82). A study conducted by Tian et al. observed a decrease in histone H3K4 methylation level and global DNA methylation in oocytes treated with a zinc deficient diet. The authors of this study also found that supplementing zinc deficient animals with methyl donors during IVM restored histone H3K4 and increased IVF success rates (87).

Regarding DNA methylation, there are limited data about the possible interplay between diet and female reproductive health. In general, nutrients modulate DNA methylation by acting as methyl donors (methionine, choline, and folate), providing co-factors essential for methyltransferases activity (vitamins B2, B6, and B12) and modulating the activity of enzymes in the methylation process (polyphenol) (161). Folic acid supplementation is recommended during pregnancy due to its prophylactic role, but its influence on women undergoing ART is not as widely recognized. One study showed that a moderate intake of folic acid is positively associated with a decreased proportion of developmentally delayed embryos in women undergoing ART (84). The authors of this study also observed that a moderate intake of folic acid in the ART population is associated with reduced DNA methylation variance in imprinting control regions [KCNQ1 overlapping transcript 1 (Kcnq1ot1), small nuclear ribonucleoprotein polypeptide N (SNRPN) and paternally expressed gene 1 (PEG1)] and increased global DNA methylation levels in both embryonic and placental tissues (84).

It has been shown that several dietary components influence the epigenome and have beneficial health outcomes. Therefore, the development of an “epigenetic diet” used to neutralize epigenomic aberrations might pave the way toward an innovative approach to improve fertility rates and female reproductive health (162).

### Microbiota in female reproduction and ART

It is well-known that dietary components influence the composition of the microbiome, however, whether this effect has an impact on female reproductive health and fertility rates is not clearly understood. Nutritional factors shape the microbiome either directly by enhancing/inhibiting microbial growth or indirectly by influencing the immune system and metabolism (163). The relationship between diet and gut microbiome and its influence on female reproductive health and ART outcomes is not clearly understood. A study conducted by Nagy et al. found that elevated levels of trimethylamine-N-oxide (TMAO) are negative predictors of oocyte fertilization and embryo quality in women undergoing IVF (77). TMAO is a diet-induced microbial metabolite that is produced from microbial utilization of dietary choline (present in fish and dairy products) and L-carnitine (present in red meat) (164). The authors reported decreased levels of TMAO and its intermediate gamma-butyrobetaine in follicular fluid of oocytes developing into top-quality embryos compared to oocytes developing into low-quality embryos. However, a non-significant association was found between levels of TMAO precursors (choline and L-carnitine) and oocyte and embryo quality (77). Another study showed the positive impact of dietary fiber intake on female infertile patients treated with ART (78). The intake of partially hydrolyzed guar gum (PHGG), a prebiotic water-soluble fiber known to modulate the gut microbiome, improved gut dysbiosis and the success rate of ART in these women. Enhanced Bifidobacterium abundance and reduced Paraprevotella and Blautia abundance were identified as potential predictive microbial markers of successful pregnancy (78).

Vaginal microbial composition is also an important indicator of female reproductive health and potential ART success. A recent prospective exploratory study suggested that identifying vaginal microbial profiles before IVF-ICSI could help to predict pregnancy outcomes and improve success rates (165). The authors showed that female patients with a non-favorable vaginal microbial profile (relative Lactobacillus load <20%, Proteobacteria >28% and presence of G. vaginalis) had seven times lower chance of pregnancy than those with a more favorable profile (high abundance of Lactobacillus crispatus and Lactobacillus iners) (165). These findings indicate a critical role for the vaginal microbiome in the female reproductive health and ART outcome. Undoubtedly the study of associations between diet, vaginal microbiome and fertility is an area of significant interest where additional research is required.

## Future perspective of personalized nutrition in ART

The use of nutraceuticals has been suggested as a potential therapeutic tool to treat women with reproductive disorders. Nutraceuticals are defined as food products that can be administered as dietary supplements with physiological benefits beyond basic nutritional needs (e.g., vitamins, minerals, herbs, fatty acids, etc.) (166). They are considered relatively safe and inexpensive compared to other chemical agents and are even thought to be effective in modifying the course of some disorders like breast cancer, cervical cancer, and ovarian cancer (167). Encouraging data support the use of nutraceuticals for female reproductive disorders. In women undergoing IVF, the intake of DHA and alpha-linoleic acid (ALA) has been positively associated with an increased number of follicles, E2 serum levels and favorable embryo quality (18). Other studies have suggested that regular intake of multivitamins and iron reduces the risk of ovulatory dysfunction (23, 28). The therapeutic effectiveness and safety of nutraceuticals depend mainly on their formulations (food extracts, isolated active constituents, etc.); thereby, quality control programs are critical to monitor their production. One main limitation identified is the absence of governmental agencies to regulate manufacturing practices and quality standards. As a result, many products invade the market with no scientific evaluation and validation. National and international policies are therefore urgently needed to standardize and validate production, and also to regulate research and development (168).

Recently, the use of probiotics in clinics has gained popularity amongst patients and general consumers due to the perception that they may modulate the microbial composition of the human body and affect disease processes (169). Gut and vaginal microbial composition are known to play a significant role in the wellbeing of the female reproductive system, which explains the growing interests in the field of human reproduction and ART (77, 78). According to the International Scientific Association of Probiotics and Prebiotics (ISAPP), a probiotic is defined as the adequate administration of living microorganisms that confer a benefit on the host (170). Probiotics have been therefore suggested as potential therapeutic tools for the healing of fertility-related disorders and enhancers of female reproductive health in general.

The oral administration of probiotics (Lactobacillus rhamnosus GR-1 and Lactobacillus fermentum RC-14) has been shown to maintain and restore normal vaginal microbial composition in patients previously diagnosed with vaginal dysbiosis (171). In addition, low colonization of Lactobacillus species was found to be associated with poor reproductive outcomes, namely implantation failure, pregnancy loss and adverse pregnancy outcomes (172–174). Additional research is needed to investigate the potential benefits of probiotics on female reproduction and to characterize strain-specific biological effects (169, 175). The pattern of probiotic administration and the identification of the target population should be taken into account when considering this therapeutic approach (176). As probiotics may affect pathophysiologic disease pathways and modulate their risk factors, the proper selection of the study population should take into account basic physiology. In addition, the use of both epidemiological background data and intervention methods may help in identifying the optimal probiotic to be used for the specific population based on their genetics, microbial profile and other clinical parameters (176).

The use of prebiotics has also been suggested as a potential therapeutic tool, as they are non-digestible carbohydrates that help probiotic microorganisms to grow and achieve their potential beneficial effect on human health. In addition, prebiotics, such as inulin and fructo-oligosaccharides, are believed to have a synergistic impact on health when combined with probiotics (177). A study by Cho et al. concluded that the intake of prebiotics (oligo-fructose) improves the pregnancy index in obese rat (178). Another study however observed no abnormalities or changes in the fertility rate and other reproductive function parameters in rats treated with prebiotics (galacto-oligosaccharides) (179). Kyno et al. found that IVF patients presenting with non-Lactobacillus-dominated microbiota (NLDM) in their endometrium had increased pregnancy rates following the restoration of their vaginal microbial flora to Lactobacillus-dominated microbiota (LDM) with the use of antibiotics and prebiotic/probiotic supplements (180). Needless to say, additional research is required to identify the true impact of prebiotics/probiotics on female reproductive health and shed light on its potential use as a therapeutic intervention.

The introduction of nutritional therapy opened the door for a new therapeutic intervention for disorders of the female reproductive system. However, there are several challenges to this end. There are no official or identified nutritional guidelines established for female fertility and related outcomes. This is likely a result of the inherent complexity in investigating the potential interplay between nutritional factors, microbial composition, genome and epigenome and its impact on female reproduction (77).

## Conclusion

**Figure 1:**
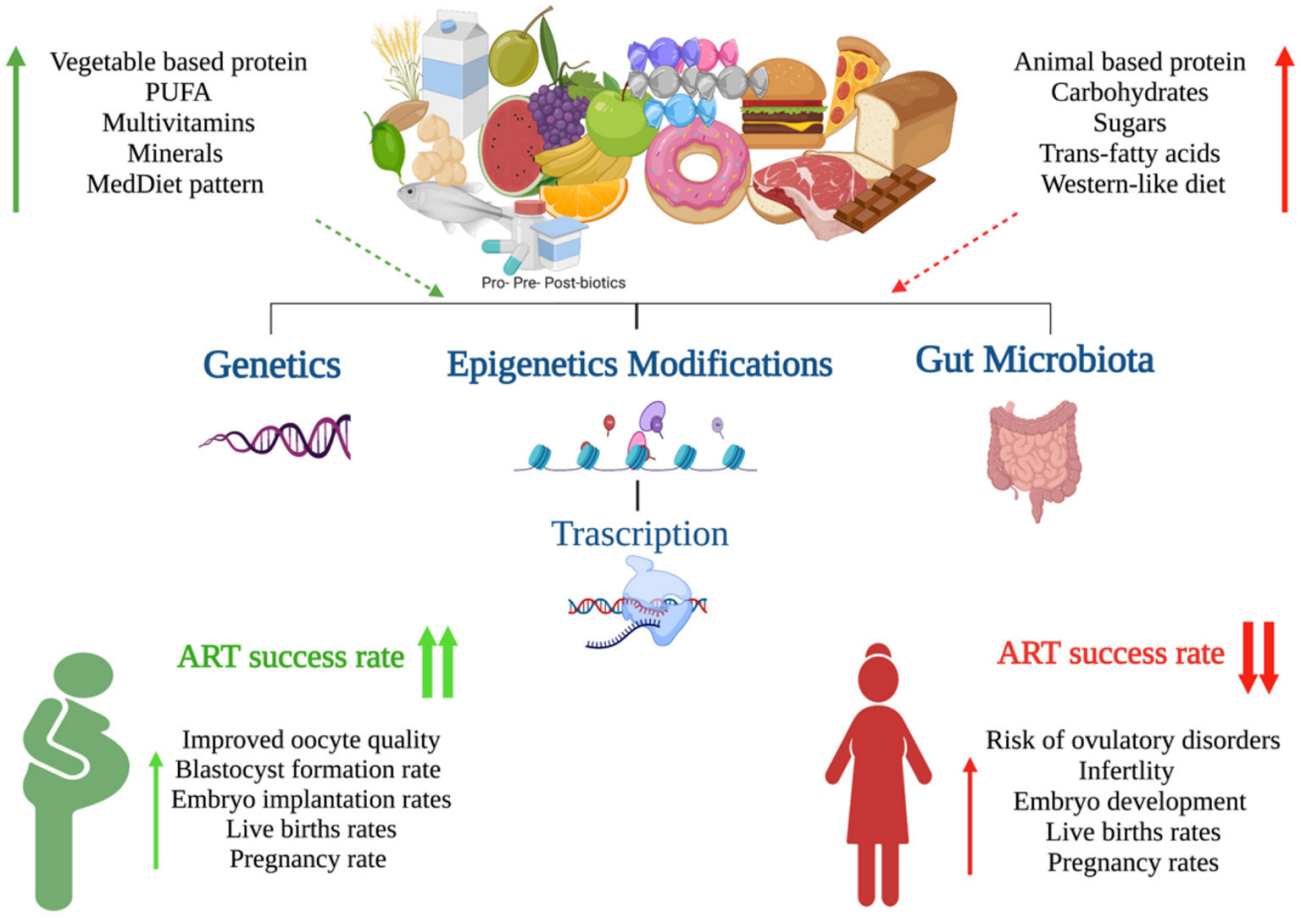
Graphical summary of the impact of nutrition on female fertility (Created with BioRender.com).

The “Food as Medicine” movement has been gaining popularity for several years. More and more people worldwide are becoming consciously aware of their nutritional habits and engage in efforts to modify their diets to reap various health benefits. In parallel, health professionals are increasingly open to the practice of “prescribing” nutritional changes or launching specialized diet programs aiming to prevent, limit or even reverse disease by changing what patients eat. In particular, couples who struggle with fertility issues and more so those who embark on ART treatments, are often highly motivated and willing to follow specialist advice especially when it comes to lifestyle changes, diet or intake of supplements, as the possibility that they will be able to overcome infertility by natural methods without the use of invasive treatments or drugs is usually very appealing to them. It does appear that nutrition plays an essential role in female reproductive health and outcomes by modulating the genome, epigenome and microbiome composition and in response to the individual genetic background. Several dietary factors, such as PUFA, folate, fiber, starch as well as the Mediterranean diet, appear to exert beneficial effects on female fertility and the success of ART (Figure 1). In contrast, other dietary factors (e.g., trans saturated fatty acids) may negatively impact female reproductive health, contributing to ovulatory disorders, reduced fecundability and endometriosis (6, 20). Understanding the direct link between dietary intake and female fertility is crucial as diet is directly implicated in the development of other chronic metabolic conditions, such as obesity, that have an impact on reproductive health. Despite the growing research interest and available evidence as discussed in our review, significant work remains to be done. A large number of relevant studies rely on relatively subjective tools and there is always the element of bias, more so in emotionally burdened infertile couples who complete specific interviews or nutritional questionnaires Fortunately, novel advances in genomic sciences are emerging which are expected to enhance our understanding of the nature and determinants of reproductive functions and the interplay with nutrition. The development of Nutrigenomics & Nutri(epi)genetics has the potential to pave the way for the adoption of precision nutrition in infertility management. Specific patients may have diverse dietary needs and tailored nutritional changes may activate relevant molecular pathways to meet the final goals of treatment Therefore, in the not-so-distant future, personalized nutritional advice and prescription of nutraceuticals for women who desire conception may become the new normal bringing a paradigm shift in the management of reproductive system disorders.

## Author contributions

AT and SC designed the content. AK and SC draft the manuscript. AT, SA, AL, SA, and JA reviewed the draft. All authors contributed to the article and approved the submitted version.

## Funding

This work was supported by Sidra Medicine funds (SDR400174).

## Conflicts of interest

The authors declare that the research was conducted in the absence of any commercial or financial relationships that could be construed as a potential conflict of interest.

## Publisher's note

All claims expressed in this article are solely those of the authors and do not necessarily represent those of their affiliated organizations, or those of the publisher, the editors and the reviewers. Any product that may be evaluated in this article, or claim that may be made by its manufacturer, is not guaranteed or endorsed by the publisher.
